# An open-source software tool for the generation of relaxation time maps in magnetic resonance imaging

**DOI:** 10.1186/1471-2342-10-16

**Published:** 2010-07-30

**Authors:** Daniel R Messroghli, Andre Rudolph, Hassan Abdel-Aty, Ralf Wassmuth, Titus Kühne, Rainer Dietz, Jeanette Schulz-Menger

**Affiliations:** 1 Cardiac MRI Unit, Franz-Volhard-Klinik, Charité University Medicine, Berlin, Germany; 2 Cardiovascular Imaging Unit, Department of Congenital Heart Defects and Pediatric Cardiology, Deutsches Herzzentrum Berlin, Germany

## Abstract

**Background:**

In magnetic resonance (MR) imaging, T1, T2 and T2* relaxation times represent characteristic tissue properties that can be quantified with the help of specific imaging strategies. While there are basic software tools for specific pulse sequences, until now there is no universal software program available to automate pixel-wise mapping of relaxation times from various types of images or MR systems. Such a software program would allow researchers to test and compare new imaging strategies and thus would significantly facilitate research in the area of quantitative tissue characterization.

**Results:**

After defining requirements for a universal MR mapping tool, a software program named MRmap was created using a high-level graphics language. Additional features include a manual registration tool for source images with motion artifacts and a tabular DICOM viewer to examine pulse sequence parameters. MRmap was successfully tested on three different computer platforms with image data from three different MR system manufacturers and five different sorts of pulse sequences: multi-image inversion recovery T1; Look-Locker/TOMROP T1; modified Look-Locker (MOLLI) T1; single-echo T2/T2*; and multi-echo T2/T2*. Computing times varied between 2 and 113 seconds. Estimates of relaxation times compared favorably to those obtained from non-automated curve fitting. Completed maps were exported in DICOM format and could be read in standard software packages used for analysis of clinical and research MR data.

**Conclusions:**

MRmap is a flexible cross-platform research tool that enables accurate mapping of relaxation times from various pulse sequences. The software allows researchers to optimize quantitative MR strategies in a manufacturer-independent fashion. The program and its source code were made available as open-source software on the internet.

## Background

Magnetic resonance (MR) imaging is a complex imaging modality, which has gained widespread use in modern medicine. Signal intensity in conventional MR images is influenced by a multitude of physical phenomena. In particular, there are three time constants describing the behavior of the net magnetization vector M in an MR experiment: 1) the longitudinal or spin-lattice relaxation time T1, describing the recovery of the M_z _component of M; 2) the transversal or spin-spin relaxation time T2, describing the decay of the M_xy _component of M; and 3) T2*, which in contrast to T2 also includes the loss of phase coherence due to field inhomogeneities and susceptibility effects. The degree to which these time constants determine signal intensity in an MR image depend on the technical parameters that are used for image acquisition [1,2]. In clinical MR imaging, all three time constants represent characteristic magnetic properties of a given tissue [3], and changes from their normal values can be used to identify pathological states of that tissue. Examples for recent research efforts include investigations into the relaxation behavior of human brain in patients with multiple sclerosis [4,5], studies of T1 and T2* changes under pharmacological stress in coronary artery disease [6], quantification of iron overload of the heart and liver in thalassaemia major [7], and analysis of myocardial fibrosis in aortic regurgitation [8].

Due to the composite nature of the MR signal, it is not possible to acquire raw images with "pure", quantifiable T1 or T2 properties in a direct fashion. In fact, to obtain pure T1 or T2 information, it is necessary to acquire a set of raw images that use varying acquisition parameters, and to perform multi-parameter curve fitting analysis on this raw data based on the mathematical functions that describe the underlying physical processes [1]. If this is done on a pixel-by-pixel basis, so called parametric "maps" can be created. These maps provide a visualization of the T1 or T2 properties in a quantitative fashion, since the signal intensity of each pixel in such a map directly reflects the relaxation time calculated (typically in milliseconds).

In the past, a number of image acquisition schemes have been developed to enable measurement and mapping of MR relaxation times. A recent offspring of these techniques is called modified Look-Locker inversion recovery (MOLLI) and opens the door for high-resolution T1 mapping in cardiac applications such as the assessment of heart muscle scarring in patients with heart attacks [9,10]. From a post-processing point of view, most of these techniques have used proprietary software programs for map reconstruction. Where mapping procedures were embedded into standard software packages for image analysis, again only specific image data were processed, and there was rarely any information available about the actual processing algorithms used. So far, both the lack of easily accessible software tools and the uncertainty about the mode of action of "black-box" software packages have posed a significant obstacle for many non-computer-expert researchers in the medical field to study relaxation time changes in diseased tissues.

The aim of our project was to provide a simple, universal software tool that can be used on multiple computer platforms to create relaxation time maps from any image data acquired with multiple mapping schemes including MOLLI [9].

## Implementation

After a survey among MR scientists at two different MR centers (Franz-Volhard-Klinik, Berlin, Germany; Leeds General Infirmary, Leeds, UK) who were asked to list desirable features of future MR mapping software, the following basic specifications were defined:

- Reading of DICOM (digital imaging and communications in medicine) image data from different MR systems

- Image registration to correct for misregistration of source images

- T1 mapping from standard inversion recovery, conventional Look-Locker or TOMROP [11], and modified Look-Locker inversion recovery (MOLLI) data sets

- T2 and T2* mapping from single-echo and multi-echo pulse sequences

- Control over basic computing parameters

- Graphical illustration of fitting results

- Export of maps in DICOM format for post-processing with conventional MR software

- Export of maps in standard graphic formats for illustration purposes.

A high-level programming language including routines for handling of complex image data and providing the option to run programs with free runtime licenses on all major computer platforms was selected (IDL 7.0, ITT Visual Information Solutions, Boulder CO, USA) [12]. A software tool named "MRmap" was created according to the specifications requested [13] (see Additional file 1). Maps are calculated pixel-by-pixel according to the selection of the user, if the type of source images is suitable for the selected technique. Validity checks include the number of source images, common field-of-views, and appropriate timing information within the DICOM headers.

### T1 mapping

T1 maps can be generated from sets of inversion recovery images (multiple series, each containing one image) with varying inversion time (TI), Look-Locker images with varying effective TI (one series containing multiple images), or MOLLI images with varying effective TI (one series containing multiple images). After sorting the images by their corresponding TI, 3-parameter curve fitting using a Levenberg-Marquardt algorithm is performed for each pixel position where signal intensity of any of the corresponding source pixels is above the user-defined noise level. The following mathematical description of the T1 behavior is used:(1)

with A representing a scaling factor for signal intensity; B reflecting the quality of the inversion (a B value of 2 × A means perfect inversion); and TI = inversion time. If standard source images with magnitude reconstruction are used, the information regarding the initial sign of the MR signal (which might have been negative since inversion recovery-based techniques are used) is lost. Therefore, MRmap performs several cycles of additional curve fittings on the whole data set [14], where each time the signs of the signal in an increasing number of leading images are switched to negative, starting with the image with the lowest TI and finishing with the last image where TI < 0.67 × limit_T1 _[ms]. Finally, the combination of signs yielding the best (= lowest) Chi value during the curve fitting process is selected and its curve fitting results are used to reconstruct the map. As a consequence of this procedure, the computing time is depending on the number of cycles necessary for the varying of the signs, which in turn is determined by the T1 limit (limit_T1_) set by the user. For Look-Locker and MOLLI data sets, where the repetitive image acquisition itself deflects the relaxation curve to be measured, the result of the curve fitting represents T1*. MRmap automatically corrects for this effect [15] using the remaining two curve fitting parameters A and B according to(2)

### T2 mapping

For T2 mapping, single-echo (= multiple series with one image each) or multi-echo (= all images within one series) data sets can be used. First, all images are sorted by their echo time (TE). Then, for each pixel set, a Levenberg-Marquardt two-parameter curve fitting [16] is performed for(3)

### T2* mapping

The same procedures (but different source images) can be used for T2* mapping. However, there is some evidence in the literature indicating that T2* accuracy might benefit from the use of a constant offset[17]. Thus, MRmap optionally calculates T2* maps by performing three-parameter curve fitting for(4)

**Figure 1:**
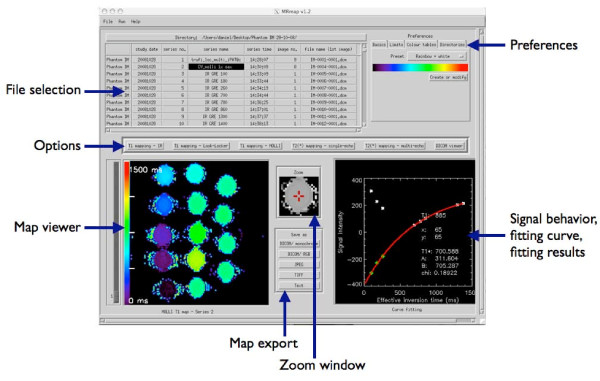
**Main screen of MRmap (screenshot with MOLLI T1 map of a gel phantom with multiple T1 times as used for assessment of software accuracy and performance)**. Elements of the graphical user interface are arranged such that workflow proceeds from top to bottom of the screen (selection of files and preferences - selection of mapping technique - output and export of results). Completed maps are presented in the map viewer. By mouse-clicking onto a pixel of interest, the surrounding region is magnified in the zoom window, and the fitting curve and fitting results of the pixel are displayed on the lower right. On this graph, signal intensities of the raw images are represented by white stars, while reconstructed (negative-signed) real data are given by green diamonds.

**Figure 2:**
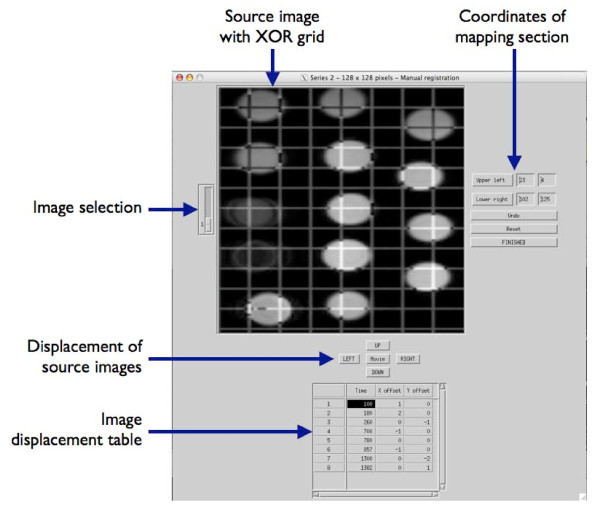
**Manual registration tool in MRmap (screenshot from MOLLI raw data acquired in a gel phantom as used for assessment of software accuracy and performance)**. Images are sorted by time (= inversion time in T1 mapping, echo time in T2/T2* mapping techniques) and can be viewed as still frame or movie. For non-static applications, images can be displaced separately by any number of pixels in x or y direction in order to correct for misregistration due to motion. A grid structure with XOR logic is superimposed onto the source images to facilitate visual registration. In order to save computation time, mapping can be confined to a sub-section of the field-of-view, which can be defined within this window or via mouse-clicking.

Figure 1 shows the main screen of MRmap as implemented according to the predefined criteria. In the upper section of the screen, essential DICOM information of the images is presented in a tabular form to facilitate selection of source images. Preferences include limits of relaxation times and noise, manual registration (Figure 2), anonymization, and color tables can be adjusted on four tabs panels. After selecting data and preferences, mapping procedures or viewing of DICOM headers of the image data can be initiated with the buttons from the bar in the center of the screen. While DICOM data will be listed in tabular form on a separate window, completed maps will be displayed on the lower left of the main screen. Mouse-clicking anywhere on the maps will show the target region with 5 fold magnification in the zoom window next to the viewer, and will produce on the lower right a graph of the raw data, together with the corresponding fitting curve and the fitting results of the pixel that was clicked upon.

The functionality of the software was tested for three different operating systems (Microsoft Windows XP pro, Apple OS X 10.5, and Fedora Linux 10) in different types of image data from multiple MR systems. Accuracy of the automated computation of relaxation times was assessed by comparing mean relaxation times of regions of interest (ROIs) from maps generated by MRmap to results from non-automated curve fitting (Prism 5, GraphPad software, La Jolla/Ca, USA) of corresponding ROIs of raw images from standardized image data sets of gadolinium-doped agarose gel phantoms with multiple T1 and T2 times (8 images per set, matrix 128 × 128, TI for IR, LL, and MOLLI: 100 to 1400 ms) acquired on a clinical 1.5 T MR system (Avanto, Siemens Medical Solutions, Erlangen, Germany). Performance of the sofware was measured in the same data sets using a 2.5 GHz Intel Core 2 Duo processor. The readability of exported maps was assessed for the following standard MR software packages: a) Mass 6.0 (Medis Medical Imaging Systems, Leiden, Netherlands), b) CMR42 (Circle Cardiovascular Imaging Inc., Calgary, Canada), c) Osirix 3.3 (Antoine Rosset, Geneva, Switzerland). MRmap and its source code were made available as open-source software under the GNU General Public License [13].

## Results

**Figure 3:**
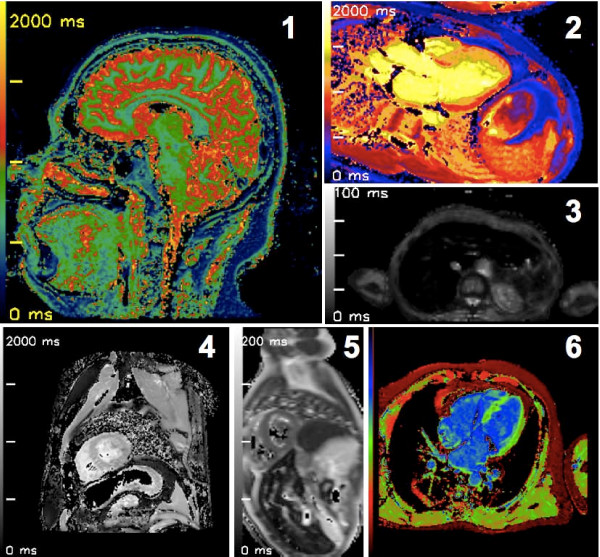
**Relaxation time maps created with MRmap from various sorts of source data**. 1) Inversion recovery T1 map; sagittal view of healthy human brain; GE 1.5 Tesla CV/i. 2) MOLLI T1 map; 3-chamber view of acute anteroseptal myocardial infarction; Siemens 1.5 Tesla Sonata. 3) Multi-echo T2* map; axial view of human liver with severe iron overload; Siemens 1.5 Tesla Avanto. 4) MOLLI T1 map; short-axis view of normal human heart; Philips 3 Tesla Intera. 5) Multi-echo T2 map; short-axis view of normal human heart; Siemens 1.5 Tesla Sonata (raw images courtesy of Dr. Taigang He, Imperial College, London, UK). 6) MOLLI T1 map; 4-chamber view; normal human heart; Philips 1.5 Tesla Intera.

**Figure 4:**
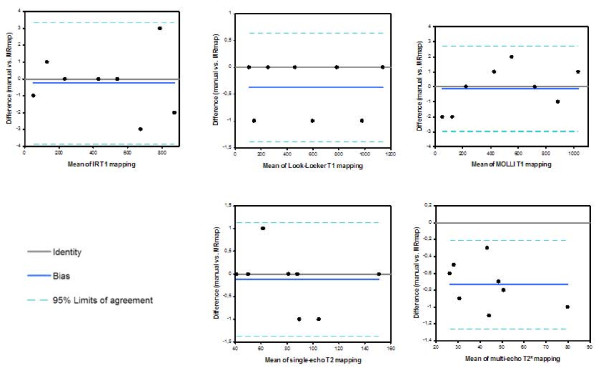
Agreement of the calculations of T1 and T2(*) times performed by MRmap as compared to non-automated ("manual") measurements (Bland-Altman plots).

**Figure 5:**
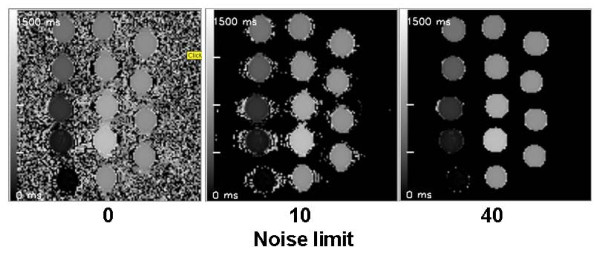
**Impact of different noise levels on T1 maps from gel phantoms**. Left panel: When all data are taken into account, there is severe background noise from the air surrounding the phantoms. Middle panel: Setting a low noise level discards virtually all of the background noise from air and only leaves noise close to the phantom borders caused by susceptibility artifacts of the phantoms. Right panel: Setting a higher noise level also removes the noise (i.e. blackens affected pixels) from susceptibility artifacts.

Figure 3 contains maps created with MRmap from various sorts of source data. Figure 4 illustrates the agreement of the calculations of T1 and T2(*) times performed by MRmap as compared to non-automated measurements. There were only minimal differences, with a small bias (0.1 ms in MOLLI to 0.74 ms in multi-echo T2*) towards underestimation of relaxation times by MRmap. Differences >1 ms were only detectable with the Look-Locker approach in a phantom with very short T1, where there were multiple pixels within the ROI without converging of the fitting algorithm due to poor quality of the raw images. MRmap automatically assigns a value of zero to such pixels, thus regions with poor fitting quality are visualized as areas of signal drop out, and ROIs of such regions might yield shorter average values. Table 1 lists the computation times of the different mapping procedures for the standardized data sets using different noise levels (Figure 5). Maps exported by MRmap in DICOM format could successfully be read and analyzed by the three software packages tested.

**Table 1:** Computation times [s] for test data sets.

	Noise level		
	0	10	40
**T1 Inversion recovery**	105	40	32
**T1 Look-Locker**	96	28	25
**T1 MOLLI**	113	36	26
T2(*) single-echo	10	5	2
T2(*) multi-echo	9	2	2

Data were acquired in gel phantoms, 8 images per set, matrix 128 × 128, T1 limit 1500, T2 limit 200) using three different noise levels (level 0: curve fitting is performed for all pixels; levels 10 and 40: pixels with signal intensities below 10 or 40 in any of the source images are excluded from curve fitting and set to zero).

## Discussion

To our knowledge, MRmap is the first open-source software tool that enables parametric T1, T2, and T2* mapping of DICOM source images on a pixel-by-pixel basis from multiple MR systems in a flexible fashion.

Other than conventional solutions that are embedded into vendor-specific application packages, the computing routines used by MRmap are well documented (see Additional file 2). Thus, results can easily be verified and do not come from a "black box", which facilitates their use for research purposes. MRmap provides a whole set of mapping routines that covers the most popular pulse sequence schemes. Our tests show that the resulting maps of the underlying relaxation times achieve the same accuracy as (tedious) non-automated curve fitting does.

By nature, the choice of a high-level graphics language as a software environment causes longer computing times than code that is written in low-level languages such as C++. This is the case in MRmap as well, where some of the mapping procedures - depending on mapping type and resolution, parameters chosen, and computer system used - can exceed one minute. However, research analyses are usually performed off-line, and therefore speed is less critical than in clinical applications. Furthermore, the exclusion of non-relevant pixels by setting appropriate noise levels allows reducing computation times drastically (see Table 1).

Apart from pure mapping procedures, MRmap provides a manual registration tool that helps to optimize mapping results if the source data contains significant motion artifacts. Image registration is a prerequisite for use in cardiac applications, where breathing artifacts are particularly common. As a limitation, MRmap in its current version does not support non-rigid registration, which can be a problem in cases where arrhythmia causes severe mis-triggering of the cardiac cycle. The integrated DICOM viewer facilitates the exploration of the source data, e.g. if images from external sites are to be analyzed. In contrast to most conventional DICOM viewers, DICOM headers from both multiple images and multiple series are listed in a tabular fashion, enabling direct comparison of header data between different images or series.

As a limitation, MRmap does not support less commonly used mapping schemes such as varying flip angle approaches [18]. These might be implemented in future versions of the software.

## Conclusion

MRmap is a flexible open-source software tool for the creation of parametric maps of MR relaxation times. Manual registration of source images, visualization of fitting results and data export in multiple image formats are supported. The software might facilitate research activities in the field of quantitative MR tissue analysis.

## Availability and requirements

Project name: MRmap

Project home page: http://sourceforge.net/projects/mrmap

Operating systems: platform independent

Program language: IDL 7.0

Other requirements: (free) IDL Virtual Machine 7.0 (or higher) [12]; X11 (on Mac OS X)

License: GNU General Public License (GPL) Restrictions to use by non-academic: MRmap is intended for research purposes only.

## Competing interests

The authors declare that they have no competing interests.

## Authors' contributions

DM designed and wrote the software, carried out the phantom tests and drafted the manuscript. AR and HAA acquired the in-vivo data and tested the software. RW participated in the design of the study and tested the software. TK, RD and JSM participated in the study design and coordination and helped to draft the manuscript. All authors read and approved the final manuscript.

## Pre-publication history

The pre-publication history for this paper can be accessed here:

http://www.biomedcentral.com/1471-2342/10/16/prepub

## Supplementary Material

Additional file 1 **.sav file of MRmap running on IDL Virtual Machine, and PDF file of MRmap manual**. Click here for file

Additional file 2 **MRmap manual**. Click here for file
