# Supplementary material for: An open-source software tool for the generation of relaxation time maps in magnetic resonance imaging
Source: BMC Med Imaging. 2010 Jul 30;10:16. doi: 10.1186/1471-2342-10-16 (PMC2919441; doi:10.1186/1471-2342-10-16)
Supplement: Additional file 1 — .sav file of MRmap running on IDL Virtual Machine, and PDF file of MRmap manual. [file 1471-2342-10-16-S1.ZIP › MRmap_v1.2_runtime/MRmap_Documentation.pdf]

MRmap v1.2

Documentation

Last updated: 22-June-2010

© Daniel Messroghli, MD
Cardiovascular Imaging Group
Congenital Heart Defects and Pediatric Cardiology
Deutsches Herzzentrum Berlin

Published under the GNU General Public License at
https://sourceforge.net/projects/mrmap

- FOR RESEARCH USE ONLY -

Contents

1 DISCLAIMER 3

2 OVERVIEW 4

2.1 Requirements 4

2.2 Features 4

2.3 Limitations, known issues 4

3 INSTALLATION 6

4 THE MAIN SCREEN 7

4.1 Menu bar 7

4.2 Preferences 7
4.2.1 Basics 8
- Images per set 8
- Registration 8
- Scaling bar 8
- Anonymous DICOM 8
- Offset correction for T2(*) 8
4.2.2 Limits 9
- T1 limit (between 500 and 3500 ms) 9
- T2(*) limit (between 50 and 3500 ms) 9
- Noise limit (between 0 and 100) 9
4.2.3 Colour tables 9
4.2.4 Directories 10
- Start directory 10
- Data export 11

4.3 File selection 11

4.4 Options 11
4.4.1 IR T1 mapping 11
4.4.2 Look-Locker T1 mapping 12
4.4.3 MOLLI T1 mapping 12
4.4.4 Single-echo T2(*) mapping 12
4.4.5 Multi-echo T2(*) mapping 12
4.4.6 DICOM viewer 13

4.5 Map viewer 13

4.6 Export 14

4.7 Fitting plots 14

5 LOG OUTPUT 15

6 MANUAL REGISTRATION 16

7 REFERENCES 18

1 Disclaimer

MRmap has been designed for research use only. It is not meant to be used for any clinical purposes or to influence any decision-making regarding medical therapies. Although a lot of effort has been taken to make MRmap as accurate as possible, the author does not take any responsibility for the correctness of any results derived by MRmap.

MRmap uses program code from DICOM_WRITER v0.21 by Bhautik Joshi (e-mail: bjoshi@geocities.com, homepage: http://cow.mooh.org) to write maps in “DICOM mode”. While maps saved in this mode should be readable by standard DICOM viewers, neither DICOM_WRITER v0.21 nor MRmap are guaranteed to write files perfectly compliant with DICOM (digital imaging and communication in medicine) standards!

If you should encounter any malfunctions or bugs, please report them at https://sourceforge.net/projects/mrmap.

This work was initially supported by a Marie Curie European Reintegration Grant from The European Commission.

2 Overview

2.1 Requirements

MRmap is written in IDL 7.0® (ITT Visual Information Solution, Boulder CO, USA) and requires a runtime license ("IDL virtual machine") that can be downloaded for free from http://www.ittvis.com/Downloads/ProductDownloads.aspx. In case of problems with registration on the IDL website, please make sure that your web browser is supported. IDL is a cross-platform application and available for Windows, Linux, Unix, and Mac OS X systems. CPU speed =1.5 GHz, memory =512 MB Ram and monitor resolution =1024x768 are recommended.

2.2 Features

MRmap was developed to allow for creating of various sorts of parametric "maps" of magnetic resonance (MR) relaxation times on a pixel-by-pixel basis from different types of MR images. This version features:

- T1 mapping from multi-experiment inversion recovery (IR)-prepared images
- T1 mapping from Look-Locker/ TOMROP type source images
- T1 mapping from MOLLI source images
- T2(*) mapping from single-echo (=multi-experiment) (turbo) spin echo (T2) or gradient echo (T2*) images
- T2(*) mapping from multi-echo (turbo) spin echo (T2) or gradient echo (T2*) images
- Manual registration of source images
- Graphical illustration of signal behaviour and curve fitting for selected pixels
- Creation and use of user-defined colour tables
- Tabular viewing of text information from DICOM headers of single or multiple studies
- Export of parametric images in DICOM-like, TIFF, JPEG, and text format

New in v1.2:

- Mapping of sub-sections of the field-of-view
- Zoom window
- Option for offset correction in T2* mapping

2.3 Limitations, known issues

MRmap is set to scan directories with up to 200 DICOM image series. If directories should contain more than 200 series, they must be split. The number of images that can be used for one parametric map is limited to 200.

It has been observed that IDL might cause program crashes if file paths become overly complicated. Therefore, it is recommended to use image directories that are located

on a high level of the directory tree and have simple names with no white space or special characters.

Similarly, file or directory names that include unusual symbols might cause crashes during file writing of MRmap on some platforms. In such cases, manual save mode should be selected and maps should be saved manually using simplified file names.

MRmap is able to perform multiple mappings in one session. However, all image data sets selected at the same time need to be of the same type of source data and have the same image matrix. If necessary, several copies of MRmap can run in parallel on one computer to enable setup of different source data or matrix types at the same time.

The 75% tick of the scaling bar sometimes is missing when a map is first displayed after processing. In these cases, forcing IDL to re-display the map by selecting the current colour table from the preferences section will help to visualize the tick.

3 Installation

After the installation of IDL/vm (see ***2.1***), an MRmap folder should be created and the MRmap program file and documentation file copied into it. IDL/vm should always be installed with root/ administrator privileges into the directory recommended by the installation program! For Windows and OS X platforms, an alias of the MRmap program file should be created on the desktop, which can be used to start MRmap by mouse-clicking onto it. On Linux platforms, an application alias should be created on the desktop (set the run command to: idl –vm=/path/MRmapVM.sav).

When started for the first time, MRmap will create a preference file named "MRmapPrefs.dat". All changes to preferences will be saved to this file.

4 The main screen

After scanning the start directory for DICOM series, MRmap will present the main screen with different sections.

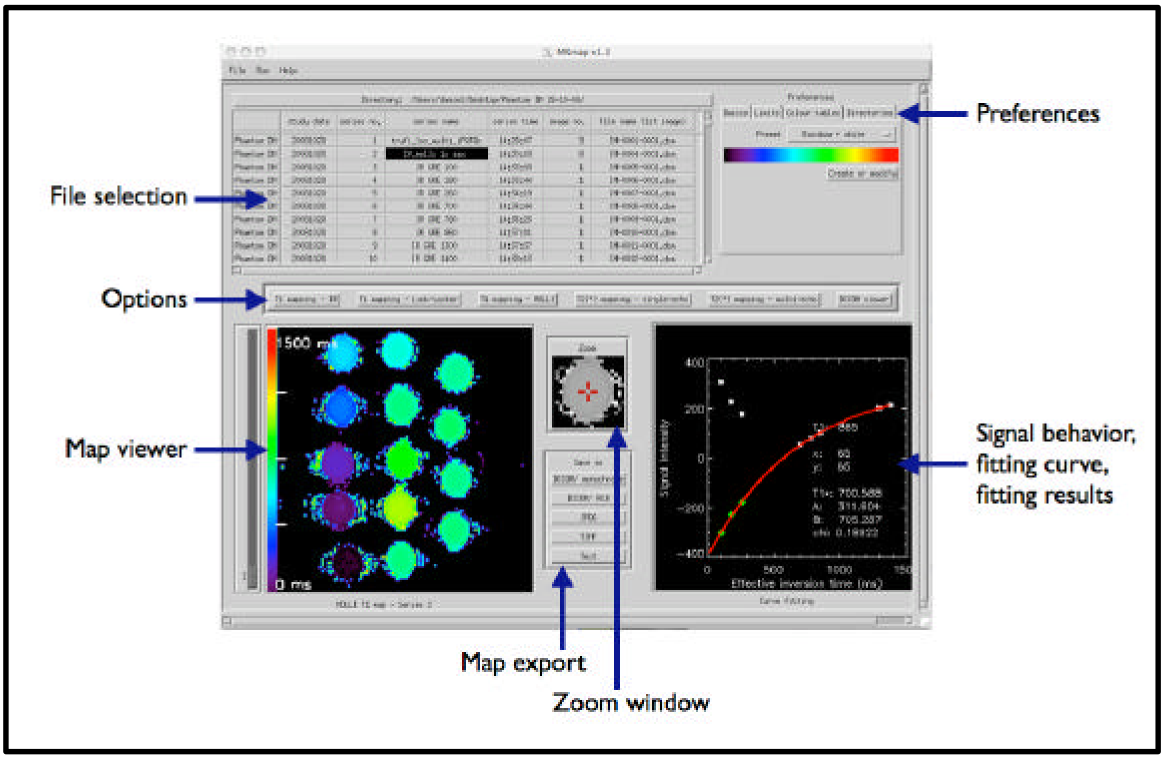

Fig. 1. Screen shot taken from the main screen of MRmap (platform: OS X 10.5). The viewer shows a MOLLI T1 map calculated from source images that were acquired in phantoms with different concentrations of Gd-DTPA. After mouse-clicking onto a pixel within the middle phantom, the region surrounding this pixel is magnified in the zoom window in the center, and the corresponding fitting curve and T1 value of the pixel are shown on the panel on the lower right.

4.1 Menu bar

The menu bar provides pull-down menus corresponding to all buttons visible on the main screen except for those in the preferences section. In addition, a help option leads to viewing of this document.

4.2 Preferences

Preferences are stored in a file named “MRmapPrefs.dat” within the MRmap directory. At first start of MRmap or if the preferences file gets lost, a new file with standard preferences will be created. The preferences section is divided into four tabs:

4.2.1 Basics

> **Images per set**

The number of source images that will be used for mapping (between 5 and 100). This figure has to match the number of images present within one series (single-experiment techniques, e.g. Look-Locker) or the number of series selected (multi-experiment techniques, e.g. IR T1 mapping), otherwise the selection will not be accepted in order to avoid processing of inadequate raw images.

> **Registration**

- "None": Computation of maps is performed directly from selected source data without further image registration.
- "Manual": Manual registration is enabled and will be started before initiation of the mapping procedure (see chapter ***5***).

> **Scaling bar**

Specifies whether a scaling bar of the selected colour table is printed on the left side of the maps along with upper and lower limits or not.

> **Anonymous DICOM**

When set to "yes", all DICOM headers of exported maps will be anonymized.

> **Offset correction for T2(*)**

When set to "yes", calculation of T2(*) is performed with offset correction according to Ghugre et al.[1] This option might be helpful in cases of very low T2* values (e.g. severe iron overload), but might result in poor fitting otherwise.

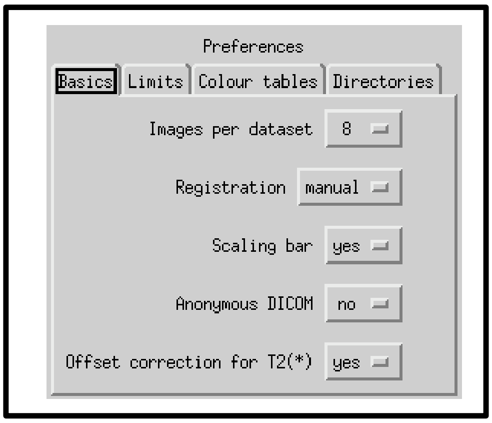

Fig. 2. Preferences section: Basics

4.2.2 Limits

> **T1 limit (between 500 and 3500 ms)**

The value selected here will be the maximum T1 value possibly given in the T1 maps (=”white” on the B-W linear colour table, top end of the scaling bar). Values above the T1 limit will be set to zero (=”black” on the B-W linear colour table, lower end of the scaling bar). The T1 limit influences the computation time because higher T1 limits require more testing for negative signs in modulus source images.

> **T2(*) limit (between 50 and 3500 ms)**

The value selected here will be the maximum T2(*) value possibly given in the T2(*) maps (=”white” on the B-W linear colour table, top end of the scaling bar). Values above the T2(*) limit will be set to zero (=”black” on the B-W linear colour table, lower end of the scaling bar).

> **Noise limit (between 0 and 100)**

The noise limit is used to avoid time consuming fitting calculations of pixels without useful signal and to clean maps from noise. Pixels whose signal intensity is below the noise limit in all source images will be skipped during curve fitting and automatically set to zero on the map.

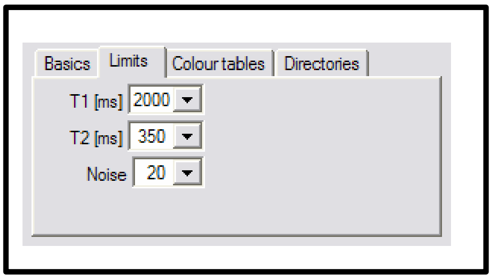

Fig. 3. Preferences section: Limits

4.2.3 Colour tables

> **Preset**

This drop list allows selecting a pre-defined colour table which will be used for display of the maps. A scaling bar of the current preset is presented underneath the drop list button. At first start, all IDL® standard colour tables are provided. In contrast to the other preferences (limits etc.), changes of the colour table preset will not only affect new maps but will also change the appearance of all maps that are still held in the map viewer. Image export in JPEG and TIFF format will maintain the selected colour table, while images saved in DICOM-like format will always use a B-W linear grey scale.

> **Create or modify**

This button enables creating new or modifying existing colour tables by calling IDL®'s XPalette routine. Amongst other features, this routine provides a help menu where in-depth information on the routine can be found. In brief, XPalette allows modifying any of the predefined colour tables provided by IDL®. When XPalette is terminated by pressing the "done" button, MRmap asks for the name of the new colour table. If the name of one of the existing colour tables is typed in, then that colour table will be overwritten by the modified colour table. Otherwise, a new colour table will be created. Pressing "cancel" will leave all colour tables unchanged.

Note: Colour tables contain 256 colours (8 bit), which will be used to cover the full range of relaxation times possible. In other words, the resulting colour of a pixel with a given relaxation time depends on its value in ms relative to the selected relaxation time limit. Example: Using the B-W linear colour table, a pixel with a T2 value of 25 ms will be lighter grey when the T2 limit is set to 100 ms than when it is set to 500 ms. Therefore, if colour tables are designed to help visually identify certain relaxation time ranges (e.g. all pixels with T1 between 100 and 200 ms should be red, any other pixels should be green and blue), then they must always be used with the same appropriate relaxation time limits in order to result in reproducible maps.

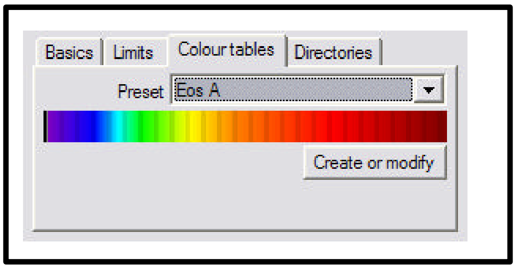

Fig. 4. Preferences section: Colour tables

4.2.4 Directories

> **Start directory**

- "Most recent": On start-up, MRmap will scan the directory that was last in use for DICOM files. If this directory should not exist any more (e.g. because it was located on a CD-ROM which is not inserted any more), then the default MRmap directory will be used.
- "Browse": On start-up, MRmap will let the user select a directory to scan for DICOM files.

> **Data export**

- "Manual": Maps will not be automatically saved after computation. All export must be performed manually (see section ***4.6***).
- "Work directory": Maps will be saved automatically within the work directory from which files were selected. This requires write permissions for this directory (not possible on CD-ROMs and DVDs)! Names will be created including patient name, series number of the first source image, and "reg" (if manually registered), followed by ".dcm" (for DICOM-like format). In the DICOM header, maps will be assigned a not-existing series number (>240) according to their type of map (e.g. 247 for multi-echo T2(*) maps).
- "Browse": Allows for specifying a directory where all maps will automatically be stored after computation (naming of maps: same as for "work directory").

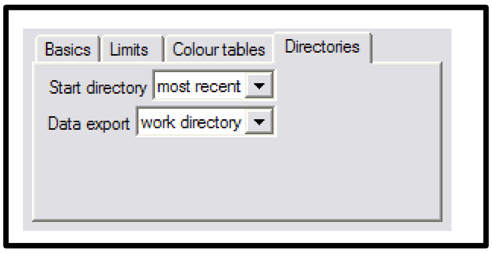

Fig. 5. Preferences section: Directories

4.3 File selection

In this area, all series of DICOM images present in the current work directory are listed in a table. The current work directory is given in the bar above the table and can be changed by mouse-clicking upon that bar. To enable any of the options given in the "options" section, series can be selected by clicking with the (left) mouse button upon any of the corresponding cells within the table. Multiple cells can be selected by holding the "shift" (for contiguous) or "strg/ctrl" button (for non-contiguous cells).

4.4 Options

Here, the following operations can be initiated if appropriate series have been selected in the file selection area.

4.4.1 IR T1 mapping

T1 maps are generated from source images acquired with multiple inversion recovery-prepared pulse sequences using different inversion times (TI). The number of series selected must be the same as specified in "images per set" (***4.2***). Each series must contain one image with a specific TI. If no inversion pulse was applied, TI is automatically set to 10000 ms (where full T1 recovery is assumed). The T1 calculations are based on a 3-parameter Levenberg-Marquardt curve fitting procedure for: y = A – B (exp-TI/T1). See also "T1 limit" (***4.2***).

4.4.2 Look-Locker T1 mapping

T1 maps are generated from source images acquired with a Look-Locker[2]/ TOMROP[3] type pulse sequence. The number of images within each series must be the same as specified in "images per set" (***4.2***). The T1 calculations are based on a 3-parameter Levenberg-Marquardt curve fitting procedure for: y = A – B exp(-TI / T1*). Correction for read-out-induced attenuation of the relaxation curve[4] is attempted by using the three curve fitting parameters T1*, A, and B for the calculation of T1:
T1 = T1* ((B / A) – 1). See also "T1 limit" (section ***4.2***).

4.4.3 MOLLI T1 mapping

T1 maps are generated from source images acquired with the MOLLI (modified Look-Locker inversion-recovery) pulse sequence. In its current version, MRmap accepts data acquired with the Philips NT 9.1 and 2.6.1 pulse sequences by Sebastian Kozerke (ETH Zürich, Switzerland) and the pre-WIP Siemens Sonata (VA 25) and WIP Siemens VB15 pulse sequences implemented by Andreas Greiser (Siemens Medical Solutions, Erlangen, Germany). The number of images within each series must be the same as specified in "images per set" (***4.2***). T1 calculations are performed as for Look-Locker images. For further details regarding the Look-Locker and MOLLI T1 calculations performed by MRmap, please see references[4-8]. See also "T1 limit" (section ***4.2***).

4.4.4 Single-echo T2(*) mapping

T2(*) maps are generated form source images acquired with multiple (turbo) spin echo (T2) or gradient echo (T2*) pulse sequences using different echo times (TE). The number of series selected must be a multiple of the value specified in "images per set" (see ***4.2***). Each series must contain one image with a specific TE (thus referred to as "single-echo"). With offset correction set to "no" (4.2.1), the T2(*) calculations are based on a 2-parameter Levenberg-Marquardt curve fitting procedure for: y = A exp(-TE / T2$^{(*)}$). With offset correction[1] set to "yes", T2(*) a 3-parameter fitting is performed for y = A exp(-TE / T2$^{(*)}$) + B . See also "T2(*) limit" (***4.2***).

4.4.5 Multi-echo T2(*) mapping

T2(*) maps are generated from source images acquired with a multi-echo (turbo) spin echo (T2) or gradient echo (T2*) pulse sequence. The number of images within each series must be the same as specified in "images per set" (see ***4.2***). Each image must have a specific TE. Multi-echo T2(*) calculations are performed exactly as single-echo calculations, using a 2-parameter Levenberg-Marquardt curve fitting procedure for: y = A exp(-TE / T2$^{(*)}$)or a 3-parameter fitting for y = A exp(-TE / T2$^{(*)}$) +B in case of offset correction set to "yes". See also ***4.2***, "T2(*) limit".

4.4.6 DICOM viewer

This option will view the DICOM text headers of single or multiple images or series in tabular form (Fig. 2). If single series are selected, headers of all images within that series will be listed next to each other. If multiple series are selected, the headers of the first image within each series will be displayed. Headers can be saved as a text file by choosing "DICOM dump/ Save as" in the menu bar of the DICOM viewer window. "DICOM dump/ Close window will close the DICOM viewer window.

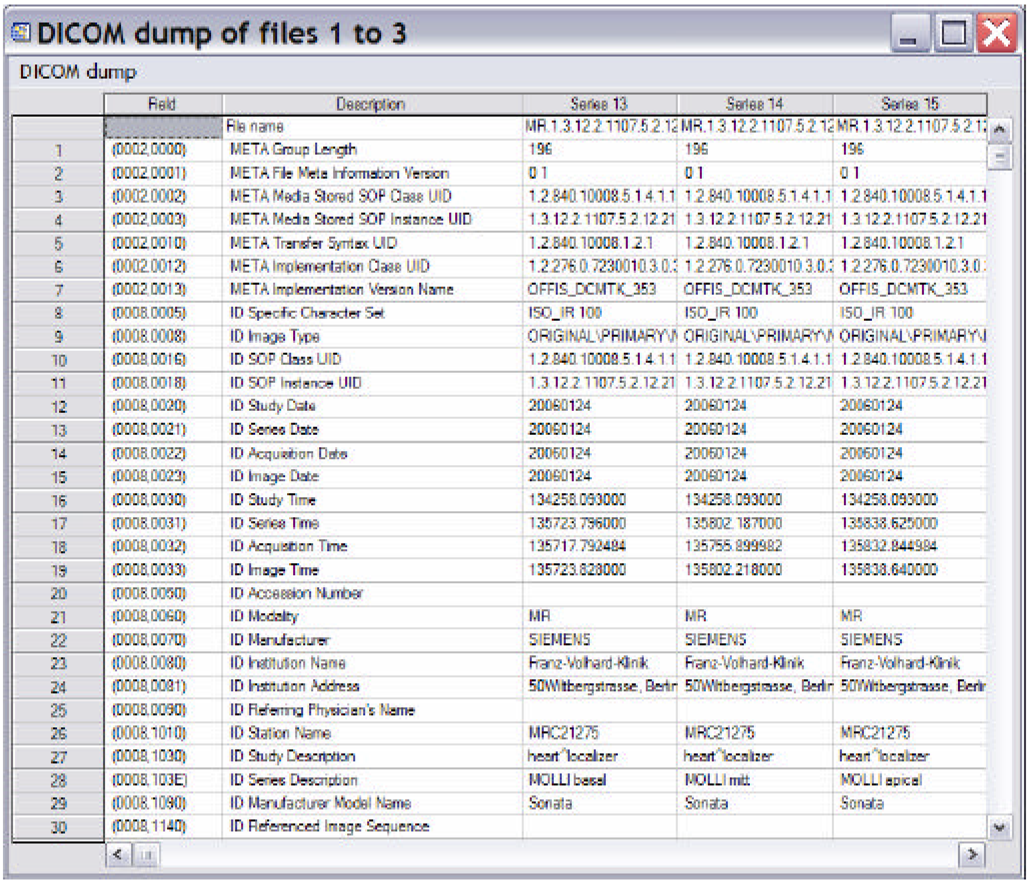

DICOM dump of files 1 to 3

DICOM dump

	Field	Description	Series 13	Series 14	Series 15
		File name	MR.1.3.12.2.1107.5.2.12	MR.1.3.12.2.1107.5.2.12	MR.1.3.12.2.1107.5.2.1
1	(0002,0000)	META Group Length	196	196	196
2	(0002,0001)	META File Meta Information Version	0 1	0 1	0 1
3	(0002,0002)	META Media Stored SOP Class UID	1.2.840.10008.5.1.4.1.1	1.2.840.10008.5.1.4.1.1	1.2.840.10008.5.1.4.1.1
4	(0002,0003)	META Media Stored SOP Instance UID	1.3.12.2.1107.5.2.12.21	1.3.12.2.1107.5.2.12.21	1.3.12.2.1107.5.2.12.21
5	(0002,0010)	META Transfer Syntax UID	1.2.840.10008.1.2.1	1.2.840.10008.1.2.1	1.2.840.10008.1.2.1
6	(0002,0012)	META Implementation Class UID	1.2.276.0.7230010.3.0.	1.2.276.0.7230010.3.0.	1.2.276.0.7230010.3.0.
7	(0002,0013)	META Implementation Version Name	OFFIS_DCMTK_353	OFFIS_DCMTK_353	OFFIS_DCMTK_353
8	(0008,0005)	ID Specific Character Set	ISO_IR 100	ISO_IR 100	ISO_IR 100
9	(0008,0008)	ID Image Type	ORIGINAL\PRIMARY\	ORIGINAL\PRIMARY\	ORIGINAL\PRIMARY\
10	(0008,0016)	ID SOP Class UID	1.2.840.10008.5.1.4.1.1	1.2.840.10008.5.1.4.1.1	1.2.840.10008.5.1.4.1.1
11	(0008,0018)	ID SOP Instance UID	1.3.12.2.1107.5.2.12.21	1.3.12.2.1107.5.2.12.21	1.3.12.2.1107.5.2.12.21
12	(0008,0020)	ID Study Date	20060124	20060124	20060124
13	(0008,0021)	ID Series Date	20060124	20060124	20060124
14	(0008,0022)	ID Acquisition Date	20060124	20060124	20060124
15	(0008,0023)	ID Image Date	20060124	20060124	20060124
16	(0008,0030)	ID Study Time	134258.093000	134258.093000	134258.093000
17	(0008,0031)	ID Series Time	135723.796000	135802.187000	135838.625000
18	(0008,0032)	ID Acquisition Time	135717.792484	135755.899982	135832.844984
19	(0008,0033)	ID Image Time	135723.828000	135802.218000	135838.640000
20	(0008,0050)	ID Accession Number			
21	(0008,0060)	ID Modality	MR	MR	MR
22	(0008,0070)	ID Manufacturer	SIEMENS	SIEMENS	SIEMENS
23	(0008,0080)	ID Institution Name	Franz-Volhard-Klinik	Franz-Volhard-Klinik	Franz-Volhard-Klinik
24	(0008,0081)	ID Institution Address	50Wiltbergstrasse, Berlin	50Wiltbergstrasse, Berlin	50Wiltbergstrasse, Berlir
25	(0008,0090)	ID Referring Physician's Name			
26	(0008,1010)	ID Station Name	MRC21275	MRC21275	MRC21275
27	(0008,1030)	ID Study Description	heart^localizer	heart^localizer	heart^localizer
28	(0008,103E)	ID Series Description	MOLLI basal	MOLLI mitt	MOLLI apical
29	(0008,1090)	ID Manufacturer Model Name	Sonata	Sonata	Sonata
30	(0008,1140)	ID Referenced Image Sequence			

Fig. 6. DICOM dump from a set of MOLLI images acquired on different short-axis levels. The table lists all text information contained in the DICOM headers of the first image within each series.

4.5 Map viewer

Completed maps will be viewed in this area. If multiple maps have been computed within one step (e.g. multiple series of MOLLI images were selected at once), the slider below can be used to switch between maps as long as no new option has been initiated. Directing to a point within a map and clicking the left mouse button will cause the program to give mapping details of the corresponding pixel of the map in the "Fitting plots" section (see **4.6**).

4.6 Export

Completed maps can be manually exported in several file formats:

- "DICOM/ monochrome" will export the currently displayed map in DICOM-like format (see 1) using standard monochrome encoding (corresponding to the standard black-white linear colour table). Other than in automatic DICOM export (see 4.2.4, "Save mode"), destination and name of the file can be manually selected.
- "DICOM/ RGB" will export the currently displayed map in DICOM-like format using RGB encoding. That means that colour tables other than black-white linear will be maintained. As a consequence, **quantitative analysis** of relaxation times via regions-of-interests (ROIs) **will not be possible** on the resulting images because each single value is split into 3 components (red, green, blue).
- "JPEG" and "TIFF" allow exporting in JPEG and TIFF format, respectively.
- "Text" will export the map data as a raw text file.

4.7 Fitting plots

This area will show detailed mapping information for any pixel within a completed map where the mouse cursor has been directed to and clicked on in the map viewer section. X and y coordinates of the selected pixel are displayed. Curve fitting parameters are given, along with a graph illustrating the result of the curve fitting procedure. In T1 mapping, measured signal intensities will be represented by white asterisks, whereas sign-reconstructed signal intensities will be shown as green diamonds.

5 Log output

At the start of MRmap, an additional window is opened where a "log book" of all activities is recorded, including time stamps for major activities. This is also the place where error messages will be issued.

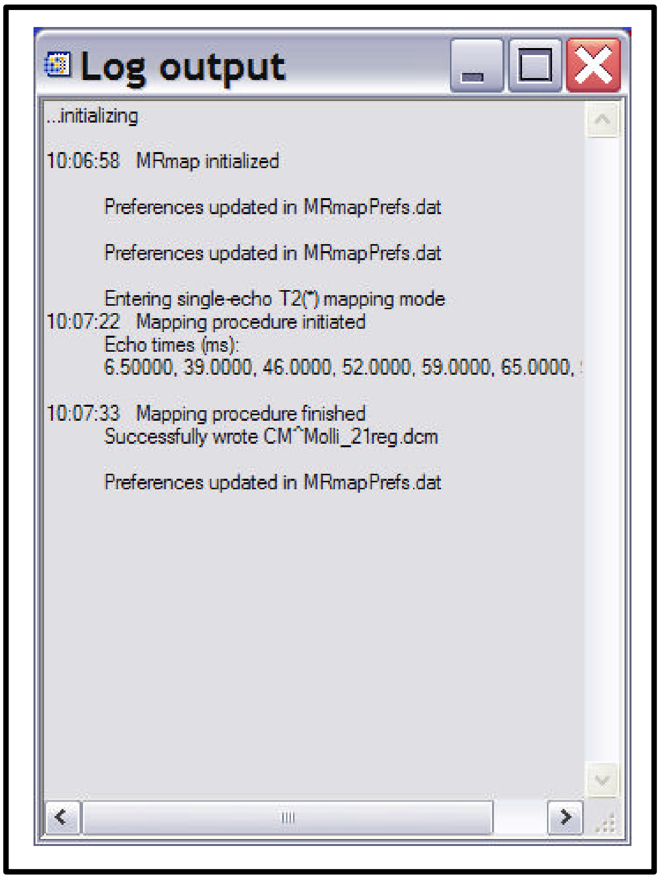

Fig. 7. Log output from an MRmap session.

6 Manual registration

If manual registration has been selected in the preferences section (see ***4.2.1***, "Registration"), a new window will appear after choosing any of the T1 or T2 mapping methods (Fig. 8). This window will allow manual registering of source images.

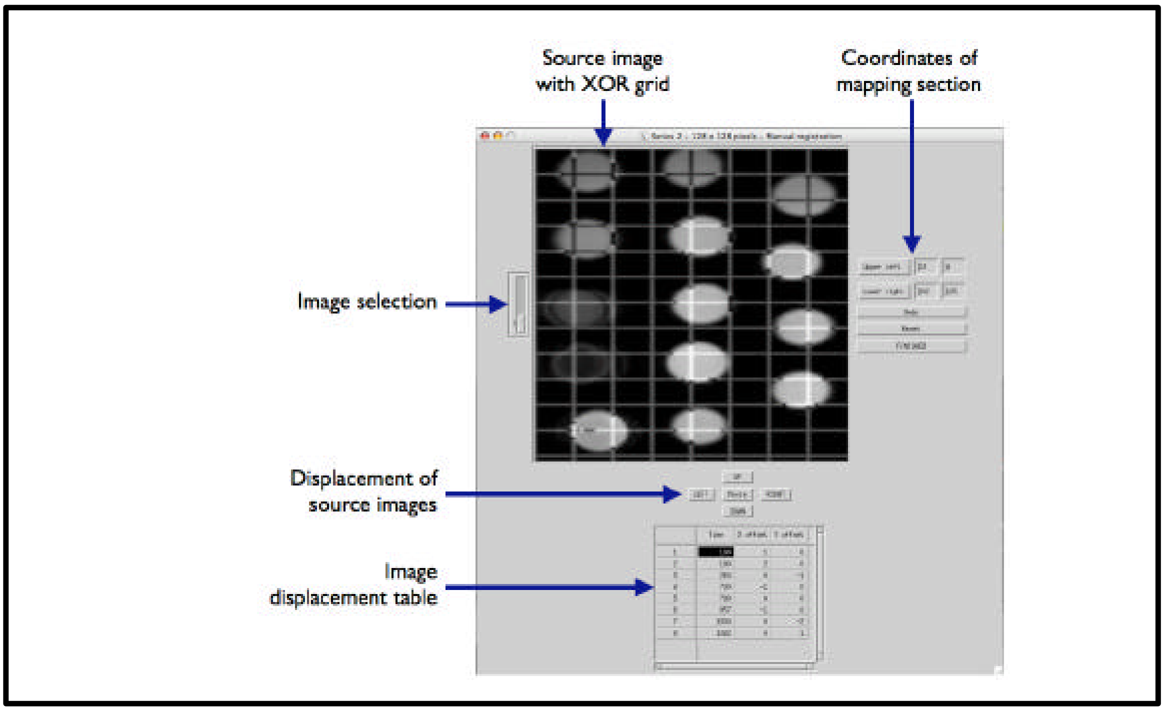

Fig. 8. Manual registration window. Screen shot from a short-axis MOLLI image set of a patient with cardiac amyloidosis.

In the upper part of the window, source images are displayed with horizontal and vertical grid lines facilitating visual localization. Images can be switched with the "up" or "down" cursor buttons or using the slider bar left to the source images. In the lower part, an offset table is presented where offsets of the source in x and y direction from their original position are listed. These offsets can be changed for the currently displayed image by pushing the "up", "down", "left" or "right" buttons in the middle part of the window. The "movie" function in the centre allows getting a quick impression of the over-all registration quality of the images. Images can also be directly selected for display by choosing a row from the offset table. In the right upper part of the window, the outer coordinates of the section of the field-of-view to be mapped are displayed (default values: full field-of-view). To change these values, they can either be directly typed into the fields for x and y of the upper left or lower right corner, or these points can be set by left (for upper left) or right clicking (for lower right) onto the image. The "undo" option allows to undo the last x or y offset operation. Pressing the "reset all" button will restore the original positions and the full field-of-view of all images. Choosing "finished" will finish the registration process of this image set, If several image sets were selected for mapping

(e.g. multiple MOLLI images sets), another registration window will open for the next image set until all sets have been registered. Finally, a pop-up window will appear and ask if the computation of the maps (which can take several minutes per map) should be started. The progress of these computations can be watched in the log output window. Note: Mapping procedures require a lot of processing capacity, which can cause some systems to seem “locked” for several minutes. This does usually not mean that the computer is crashed!

7 References

1. Ghugre NR, Enriquez CM, Coates TD, Nelson MD, Jr., Wood JC. Improved R2* measurements in myocardial iron overload. *J Magn Reson Imaging*. 2006;23:9-16.
2. Look DC, Locker DR. Time saving in measurement of NMR and EPR relaxation times. *Rev Sci Instrum*. 1970;41:250-251.
3. Graumann R, Barfuß H, Hentschel D, Oppelt A. TOMROP: eine Sequenz zur Bestimmung der Längsrelaxationszeit T1 in der Kernspintomographie. *electromedica*. 1987;55:67-72.
4. Deichmann R, Haase A. Quantification of T1 values by SNAPSHOT-FLASH NMR imaging. *J Magn Reson*. 1992;96:608-612.
5. Messroghli DR, Radjenovic A, Kozerke S, Higgins DM, Sivananthan MU, Ridgway JP. Modified Look-Locker inversion recovery (MOLLI) for high-resolution T1 mapping of the heart. *Magn Reson Med*. 2004;52:141-6.
6. Nekolla S, Gneiting T, Syha J, Deichmann R, Haase A. T1 maps by K-space reduced snapshot-FLASH MRI. *J Comput Assist Tomogr*. 1992;16:327-32.
7. Messroghli DR, Plein S, Higgins DM, Walters K, Jones TR, Ridgway JP, Sivananthan MU. Human Myocardium: Single-Breath-hold MR T1 Mapping with High Spatial Resolution--Reproducibility Study. *Radiology*. 2006;238:1004-12.
8. Messroghli DR, Greiser A, Frohlich M, Dietz R, Schulz-Menger J. Optimization and validation of a fully-integrated pulse sequence for modified look-locker inversion-recovery (MOLLI) T1 mapping of the heart. *J Magn Reson Imaging*. 2007;26:1081-1086.
